# Comparison of Procedural Sequences in Sedated Same-Day Bidirectional Endoscopy with Water-Exchange Colonoscopy: A Randomized Controlled Trial

**DOI:** 10.3390/jcm11051365

**Published:** 2022-03-02

**Authors:** Yu-Hsi Hsieh, Malcolm Koo, Chih-Wei Tseng

**Affiliations:** 1 Division of Gastroenterology, Department of Internal Medicine, Dalin Tzu Chi Hospital, Buddhist Tzu Chi Medical Foundation, Chiayi 622401, Taiwan; hsieh.yuhsi@msa.hinet.net; 2 School of Medicine, Tzu Chi University, Hualien City 970374, Taiwan; 3 Graduate Institute of Long-Term Care, Tzu Chi University of Science and Technology, Hualien City 970302, Taiwan; m.koo@utoronto.ca; 4 Dalla Lana School of Public Health, University of Toronto, Toronto, ON M5S 1A1, Canada

**Keywords:** water exchange, bidirectional endoscopy, colonoscopy, cecal-intubation time, adenoma-detection rate

## Abstract

Background: Previous studies have favored esophagogastroduodenoscopy (EGD) followed by colonoscopy as the optimal sequence in bidirectional endoscopy (BDE) with air insufflation. However, the optimal sequence in same-day BDE with WE colonoscopy is unclear. Methods: A total of 200 patients undergoing BDE with propofol sedation from May 2018 to January 2021 were randomized to either the EGD-first group (n = 100) or the colonoscopy-first group (n = 100). Results: The EGD-first group required a longer cecal-intubation time (median 16.0 min vs. 13.7 min, *p* < 0.001) and a lower Boston Bowel Preparation Scale score (8.5 vs. 9, *p* = 0.030) compared with the colonoscopy-first group. However, the EGD-first group needed a significantly lower dose of propofol (200 mg vs. 250 mg, *p* < 0.001) and a shorter recovery time (7 min vs. 13.5 min, *p* < 0.001), resulting in a shorter turnover time of the endoscopy room (39.5 min vs. 42.6 min, *p* = 0.004). There were no differences in the sedation-related adverse events, patients’ satisfaction scores, adenoma-detection rates, or the outcomes of EGD between the two groups. Conclusions: During propofol-sedated BDE, EGD followed by WE colonoscopy was more efficient with a shorter turnover time despite a longer cecal-intubation time (NCT03638713).

## 1. Introduction

Same-day bidirectional endoscopy (BDE) with esophagogastroduodenoscopy (EGD) and colonoscopy are commonly performed to evaluate gastrointestinal conditions, such as active gastrointestinal bleeding, iron-deficiency anemia, positive fecal occult blood test, and abdominal pain [1,2,3]. In addition, many asymptomatic patients undergo BDE for physical checkups or cancer screening [4]. The benefits of same-day BDE include excellent diagnostic yield, a shorter hospital stay, reduced medical costs, and quicker healthcare decision making without an increase in risk [5,6]. When both EGD and colonoscopy were indicated, about 65% of patients underwent same-day BDE in the United States [1].

Traditionally, air insufflation is used to open the lumen of the colon during colonoscopy. Multiple studies have been performed to determine the optimal sequence (i.e., EGD-first or colonoscopy-first) of same-day BDE using air insufflation for colonoscopy [4,5,7,8,9,10,11,12]. Most of them favored the EGD-first sequence because of the lower sedative requirement [4,5,7,8,10,13], the faster recovery [4,5,8], and the reduced patient discomfort during EGD [8,11,12] compared to the colonoscopy-first sequence.

Water exchange (WE) is a well-establish insertion method for colonoscopy [14,15,16]. A recent modified Delphi review confirmed that WE could increase adenoma-detection rate (ADR) and reduce insertion pain compared to gas (air or CO_2_) insufflation [17]. WE has also been shown to increase adenoma detection in propofol-sedated patients [18]. WE colonoscopy is characterized by advancing the scope in an airless lumen guided by the infusion of water, which is immediately aspirated to keep the colon lumen collapsed, aiming for the almost complete removal of infused water upon reaching the cecum [17]. Another distinctive feature of WE is the removal of air pockets and fecal debris during insertion in order to achieve better bowel cleanliness. However, performing EGD before WE colonoscopy could also be problematic. A gastroscope can generate a maximum air-flow rate of up to 2 L/min without resistance and achieve overpressures of up to 45 kPa (kilopascal) if there is inadequate air drainage [19]. The air insufflated during EGD may be passed into the intestine and subsequently the colon, carrying the intestinal contents along with it, which might make WE difficult.

To the best of our knowledge, there have been no studies on the impact of performing EGD prior to WE colonoscopy. Therefore, we conducted this prospective randomized controlled trial to evaluate the performance of WE colonoscopy before and after EGD. We tested the hypothesis that EGD preceding WE colonoscopy could prolong the cecal-intubation time. We also evaluated other procedural outcomes to determine the optimal sequence of BDE with WE colonoscopy.

## 2. Materials and Methods

### 2.1. Study Population

This prospective, randomized controlled trial was conducted between May 2018 and January 2021 at Dalin Tzu Chi Hospital, Buddhist Tzu Chi Medical Foundation, Chiayi, Taiwan. We included patients aged 20 to 80 years who underwent sedated BDE in the physical-examination department. The exclusion criteria included patients who were <20 years old or >80 years old, a lack of standard bowel preparation, known obstructive lesions of the gastrointestinal tract, an allergy to propofol, undergoing hemolysis, American Society of Anesthesiology Risk Class 3 or higher, with a history of partial colectomy, or refusal to provide written informed consent. The study was approved by the Institutional Review Board of Dalin Tzu Chi Hospital (Approval Number: A10604001) and was registered with ClinicalTrials.gov (NCT03638713). All patients signed informed consent forms.

### 2.2. Randomization and Allocation

A total of 200 patients were allocated to one of two groups (EGD-first or colonoscopy-first) by computerized randomization. These codes were contained in prearranged opaque envelopes, which were opened immediately before the BDE. Patients in the colonoscopy-first group received colonoscopy followed by EGD and those in the EGD-first group received EGD followed by colonoscopy. In both groups, colonoscopy was performed with the WE method [20,21]. Warm-to-the-touch tap water was infused through the accessory channel of the colonoscope using a foot-switch-controlled water pump (OFP-2, Olympus, Tokyo, Japan). Air insufflation was used during the withdrawal phase after the cecum was reached in both groups.

### 2.3. Preparation and Procedure

An assistant nurse obtained the demographic data (age, sex, height, and weight), history of abdominal or pelvic surgery, constipation, chronic use of laxatives, and history of drinking or smoking before BDE. Two board-certified endoscopists (CWT and YHH) with an experience of 2000 to 3000 WE colonoscopies performed the exam using a standard upper endoscope (GIF-Q-260; Olympus Optical Co., Ltd., Tokyo, Japan) and colonoscope (CF-Q-260 or CF-HQ-290; Olympus Optical Co., Ltd., Tokyo, Japan).

All patients received a standard split-dose bowel preparation with 2 L of polyethylene-glycol solution (Klean Prep solution; Helsinn Birex Pharmaceuticals Ltd., Dublin, Ireland) plus 10 mg bisacodyl. Immediately before BED, all patients ingested 15 mL of dimethylpolysiloxane (Yungshin Pharmaceutical, Taichung, Taiwan) and received 10 puffs of local xylocaine spray (Xylocaine 10% Spray, AstraZeneca AB, Södertalje, Sweden) (1 puff = 10 mg of lidocaine). Scopolamine-N-butylbromides were not administered.

In both groups, propofol (Diprivan, AstraZeneca, Stockholm, Sweden) was administered for sedation. After an initial bolus of 1 mg/kg, or 0.5 mg/kg for patients over 65 years of age, propofol was titrated in 20–30 mg increments to achieve deep sedation. The procedure started when the patient was asleep, not responding to repeated name calling but ventilating spontaneously. Additional propofol was also given whenever the patient had pain responses (moans, grimaces, and movements). If the patient woke up after the first endoscopy, additional propofol would be added to achieve adequate sedation for the second endoscopy. The dose of propofol administered during the BDE was recorded. During the procedure, the electrocardiogram, blood pressure, and oxygen saturation of the patient were monitored.

All procedures started with the patient in the left lateral decubitus position. During the interval between the two endoscopy procedures, we adjusted the position of the examination bed and changed the endoscopes. During colonoscopy, we implemented maneuvers to avoid and reduce loops in all patients when the tip of the scope moved paradoxically or did not advance. Intubation of the cecum was defined as successful when the base of the cecum could be touched by the tip of the colonoscope, which was confirmed by identifying the appendiceal orifice and/or the ileocecal valve. Detailed observations were made during the withdrawal phase. During the EGD examination, the gastroscope was advanced from the oral cavity to the second portion of the duodenum. Systemic observation followed by photo documentation was performed. After the completion of BDE, patients were wheeled to the recovery room. The recovery of the patients from sedation was assessed after the procedure using the Aldrete score [22]. The score ranges from 0 to 10, and ideally, a patient should be discharged when the score is 10.

### 2.4. Study Endpoints

The primary outcome was the cecal-intubation time. The withdraw time and procedure time for colonoscopy, the procedure time for EGD, and the total procedure time for BDE were also recorded. The withdrawal time was divided into inspection time, polypectomy time, and cleaning time. The recovery time was the interval between the arrival at the recovery room and the discharge of the patient. The turnover time was the sum of the total procedure time of BDE and the recovery time. A difficult colonoscopy was defined as any colonoscopy with a cecal-intubation time of >18 min.

After completion of the colonoscopy, the quality of bowel cleansing was assessed with the Boston Bowel Preparation Scale (BBPS), a previously validated bowel-preparation scoring system based on the summation of the preparation scores from three segments of the colon (right colon, transverse colon, and left colon). The amount of water infused and aspirated during the insertion phase of the colonoscopy, use of abdominal compression, need for a change of position, and ADR were recorded. After the EGD procedure, the endoscopist was asked to rate whether the examination was adequate. The procedure would be considered adequate if four areas (esophagus, stomach, duodenum to the second portion, and retroflex for proximal stomach) were adequately examined (0: incomplete study of all four areas; 4: complete study of all four areas). At the end of the BDE, the following sedation-related adverse events were recorded: (a) hypotension: systolic blood pressure <90 mmHg; (b) hypertension: systolic blood pressure >180 mmHg for more than 60 s; (c) oxygen desaturation: <90%; (d) tachycardia: >120 heart beats per min; and (e) bradycardia: <60 heart beats per min.

A trained research assistant who was unaware of the randomization status administered a questionnaire to the patients after recovery from sedation. Patients were asked to fill a standard questionnaire using a 10 cm visual analog scale to rate the pain, bloating, nausea, dizziness, sore throat, and overall satisfaction with the procedure. The patients were also asked whether they were willing to undergo the procedure in the same manner in the future if necessary.

### 2.5. Statistical Analysis

A pilot study showed that the cecal-intubation time was 15.6 min in the colonoscopy-first group and 18.4 min in the EGD-first group with a standard deviation (SD) of 6.0 min. Using G*Power (version 3.1.9.4, Heinrich-Heine-Universität Düsseldorf, Düsseldorf, Germany), the Student’s *t*-test based on our pilot data yielded a sample size of 98 per group (a total of 196) for a significant difference at the 0.05 level with a power of 0.90. Statistical analysis was performed using IBM SPSS Statistics for Windows, Version 19.0 (IBM Corp., Armonk, NY, USA). Categorical variables were expressed as frequency count and percent of total. The chi-square test or Fisher’s exact test was used for comparison of categorical data, as appropriate. Continuous variables were described as medians and interquartile range (IQR). The Student’s *t*-test was used to compare continuous variables with a normal distribution and the Mann–Whitney U test was used for continuous variables with a non-normal distribution. A *p* value < 0.05 was considered significant.

## 3. Results

### 3.1. Patient Characteristics

**Figure 1:**
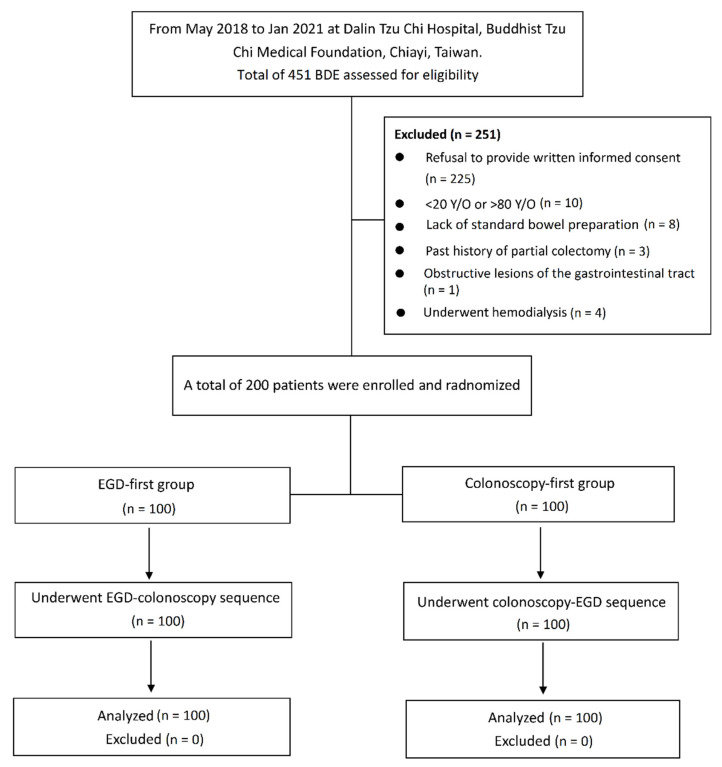
Flow chart of enrollment and intervention allocation (EGD, esophagogastroduodenoscopy; BDE, bidirectional endoscopy).

The flow diagram of enrollment, intervention allocation and exclusions is shown in Figure 1. Of the 451 patients potentially eligible for enrollment, 251 patients were excluded from the study based on the exclusion criteria. Eventually, a total of 200 patients were enrolled, with 100 patients in each group. There were 81 men and 119 women with a median age of 60 years [IRQ, 53–67 years]. Table 1 shows the baseline characteristics of the patients in the two groups. There was no significant difference in age, sex, body-mass index, history of abdominal or pelvic surgery, constipation, chronic use of laxatives, and history of alcohol drinking or smoking. A total of 37 patients underwent screening colonoscopy and 163 patients underwent surveillance colonoscopy for a heath checkup in an inpatient setting.

### 3.2. Outcomes of Bidirectional Endoscopy

The cecal-intubation time (median [IQR], 16.0 [12.1–19.1] min vs. 13.7 [11.3–16.2] min, *p* < 0.001), the procedure time of colonoscopy (26.5 [20.4–27.0] min vs. 24.0 [20.4–27.1] min, *p* = 0.003), and the total procedure time of BDE (33.1 [27.9–36.7] min vs. 30.6 [27.2–33.5] min, *p* = 0.004) were significantly longer in the EGD-first group than in the colonoscopy-first group. More patients had a difficult colonoscopy in the EGD-first group than in the colonoscopy-first group (30% vs. 10%, *p* = 0.001). The volume of water infused (1040 [750–1272] vs. 945 [750–1115] mL, *p* = 0.038) and aspirated (1100 [900–1500] vs. 1000 [705–1300] mL, *p* = 0.021) during insertion was higher in the EGD-first group than in the colonoscopy-first group. Despite using more water, the EGD-first group had a lower total BBPS score than the colonoscopy-first group (8.5 [8.0–9.0] vs. 9.0 [8.0–9.0], *p* = 0.030). Other procedure outcomes, including cecal-intubation success, ADR (45.0% vs. 49.0%, *p* = 0.571), polyp-detection rate, number of cases requiring abdominal compression, and position change were comparable between the two groups (Table 2). The procedural outcomes of EGD, including endoscopic findings, patient tolerance, and the technical adequacy were also similar between the two study groups (Table 2).

### 3.3. Propofol Doses, Recovery Time, and Sedation-Related Outcomes

The total dose of propofol used during BDE was significantly lower in the EGD-first group than in the colonoscopy-first group (200.0 [180.0–250.0] mg vs. 250.0 [200.0–300.0] mg, *p* < 0.001) (Table 3). The difference mainly came from the significantly lower loading dose before the second procedure (0 (0–20) vs. 50 (0–77.8) mg, *p* < 0.001). The recovery time (7 [4.0–10.7] min vs. 13.5 [10.0–17.0] min, *p* < 0.001) and turnover time of the endoscopy room (39.5 [34.7–45.5] min vs. 42.6 [38.3–49.3] min, *p* = 0.004) were significantly shorter in the EGD-first group than in the colonoscopy-first group. The proportions of patients who experienced oxygen desaturation, hypotension, hypertension, tachycardia, and bradycardia were similar between the two groups.

### 3.4. Patient’s Assessment of Discomfort and Satisfaction

Patients’ assessment of discomforts and satisfaction are shown in Table 3. The pain score [0 (0–1) vs. 0 (0), *p* = 0.004] and bloating score [0 (0–2) vs. 0 (0), *p* = 0.006] at discharge were significantly higher in the EGD-first group than in the colonoscopy-first group. Patients reported similar nausea, dizziness, and sore-throat scores before discharge between the two study groups. The satisfaction score and willingness to repeat BDE in the same sequence if needed were also comparable.

## 4. Discussion

To the best of our knowledge, this is the first randomized controlled trial comparing the procedure sequence in patients undergoing deeply sedated BDE with WE colonoscopy. We found that the EGD-first group had a longer cecal-intubation time and total procedure time, but lower BBPS scores as compared with the colonoscopy-first group. However, the EGD-first group required a lower dose of propofol and a shorter recovery time, resulting in a shorter turnover time.

Previous studies on the optimal sequence of BDE using air-insufflated colonoscopy did not show a significant difference in the cecal-intubation time or total procedure time of colonoscopy [4,7,10,11,12,13]. In contrast, the current study showed that the EGD-first group had a longer cecal-intubation time and total procedure time. The variation could be the result of the different insertion method of WE from that of air insufflation [17]. WE mandates the removal fecal debris and air bubbles during insertion, while during air insufflation the act of cleaning is usually performed during withdrawal. Therefore, the air insufflated during the preceding EGD should have little impact on insertion with air-insufflation colonoscopy, but a detrimental effect on WE insertion. In the current study, the EGD-first group not only required a longer cecal-intubation time, but also had lower BBPS scores despite a larger volume of water being used to remove the fecal material. The longer insertion time and lower BBPS scores in the EGD-first group supported the hypothesis that the air insufflated during EGD may be passed into the colon, carrying the intestinal contents along with it. The EGD-first group also had numerically lower ADRs than the colonoscopy-first group, but the difference was not statistically significant (45.0% vs. 49.0%, *p* = 0.571). Although a high-quality bowel cleansing was associated with a higher ADR than adequate cleansing [23], whether the worse bowel preparation in the EGD-first group would translate into a lower ADR remains to be studied.

Our current study showed that the EGD-first group required fewer doses of propofol during BDE than the colonoscopy-first group. Previous randomized controlled trials and meta-analyses on BDE with moderate or deep sedation also demonstrated that the EGD-first sequence required fewer doses of sedatives [4,5,7,8,10,13,24]. Two previous randomized controlled trials on propofol-sedated BDE proved that less propofol was required when EGD was performed first [4,12]. However, the reason why the EGD-first sequence needed less propofol than the colonoscopy-first sequence was previously unknown. In the current study, we looked at the step-by-step doses administered through the whole BDE procedure and found that the difference in the dose of propofol mainly involves the lower loading dose of the second procedure. Although the half-life of propofol is only about 2 min, its half-life in the brain is about 9 min [25,26], which is longer than the procedure time of most EGDs. Thus, patients in the EGD-first group were still sedated after EGD and required a lower dose of propofol during the following colonoscopy. On the other hand, the duration of the colonoscopy was much longer than the EGD, and usually no additional propofol was added during the withdrawal phase. As a result, in the colonoscopy-first group, most patients were partially awake after the colonoscopy and required a larger dose of propofol before EGD.

Given the lower dose of propofol used in the EGD-first group, the recovery time was shorter, as expected, which was consistent with the findings in the literature [4,5,8]. The EGD-first group had a longer total procedure time but shorter recovery time; the sum of the two (i.e., the turnover time) was shorter than the colonoscopy-first group. It appears that EGD followed by WE colonoscopy is the preferred sequence for same-day sedated BDE in terms of the efficiency of the endoscopy unit.

The pain score and bloating score before discharge were slightly but significantly higher in the EGD-first group than in the colonoscopy-first group. Only a few studies on BDE reported the discomfort scores that were evaluated before discharge of the patients [4,5,24] (Supplementary Table S1). Most of them revealed numerically higher but not statistically significantly discomfort scores in the EGD-first group. Jowhari et al. showed that the EGD-first group had a higher mean aggregated patient-discomfort score than the colonoscopy-first group (12.8, SD 9.1 vs. 10.8, SD 5.43) [24]. Hsieh et al. also reported a numerically higher post-procedure discomfort score in the EGD-first group (mean [95% confidence interval], 1.3 (0.0–5.5) vs. 0.8 (0.0–3.9), *p* = 0.104) [4]. The report of Cao et al. demonstrated a very low proportion of patients with post-procedural discomfort (EGD-first vs. colonoscopy-first: 0.94% vs. 0%) [5]. Post-colonoscopy abdominal pain was associated with the female sex, older age, an unsedated procedure, a longer procedure time, the use of air instead of carbon dioxide (CO_2_) and the timing of assessment [24,27,28]. Although a recent meta-analysis [13] reported lower discomfort scores after both procedures in the EGD-first group, the conclusion could be misleading because the analysis mostly included studies evaluating discomfort during the procedures, and the lower discomfort score actually occurred during EGD, which was correctly reported by another meta-analysis [7] (Supplementary Table S1). In the current study, the less sedation used in the EGD-first group caused less recovery time and might have further contributed to the higher pain and bloating scores. Future studies are needed to ascertain the factors associated with post-BDE discomfort. Despite the slight differences in the pain and bloating scores before discharge, the patients reported similar satisfaction scores and a willingness to repeat.

To determine the optimal sequence for BDE, a comprehensive consideration of all aspects of the procedure is needed, which should include the quality of examination, adverse effects, as well as the procedure time. In our study, the quality of the colonoscopy (including cecal intubation rate and ADR), the quality of EGD (including technique adequacy and endoscopic findings) and sedation-related adverse events were comparable between the two groups. Although the EGD-first group required a longer cecal-intubation time in the current study, it had a lower propofol dose, a faster recovery, as well as a shorter turnover time, which are key factors to an efficient operation of the endoscopy unit.

The current study had some limitations. First, the endoscopists performing the BDE were unblinded to the procedural sequence, which might lead to potential bias. Second, only two endoscopists from a single center participated in this study. The results need external validation with more endoscopists, preferably from multiple sites. Third, air insufflation was used in the current study. CO_2_ is absorbed more quickly than air from the bowel [29] and has been studied for insufflation during BDE [24]. Whether the use of CO_2_ insufflation during EGD before WE colonoscopy would result in a longer cecal intubation warrants further investigation. Fourth, for patients undergoing unsedated DBE with EGD preceding WE colonoscopy where the prolonged cecal-intubation time would not be offset by the shorter recovery time from the use of sedatives, the colonoscopy-first approach should be considered.

Despite these limitations, this study has several strengths. First, this was the first study focusing on the impact of performing EGD prior to WE colonoscopy. Second, the randomized controlled design and the appropriately estimated sample size minimized the bias and provided reliable results. Third, the study design incorporated a comprehensive evaluation of all aspects of the BDE procedure. Fourth, the step-by-step analysis of the sedation dose in the current study helps provide insight into why the colonoscopy-first group may require more sedation and recovery time, which has been widely reported but insufficiently explained in the literature.

## 5. Conclusions

The current randomized controlled trial demonstrated that EGD-first BDE with WE colonoscopy had a longer cecal-intubation time and a worse salvage-cleansing effect compared with colonoscopy-first BDE. However, patients undergoing EGD before colonoscopy required a lower dose of propofol and recovered faster, resulting in a shorter turnover time. Therefore, the EGD-first sequence is more efficient than the colonoscopy-first sequence for patients undergoing deep-sedated BDE with WE colonoscopy.

## Figures and Tables

**Table 1:** Demographic characteristics of the patients undergoing full sedation bidirectional endoscopy (N = 200).

Variable	EGD First (*n* = 100)	Colonoscopy First (*n* = 100)	*p* Value
Male, *n* (%)	38 (38)	43 (43)	0.471 ^†^
Age, median (IQR), years	59.5 (54.0–67.8)	61.0 (53.0–67.0)	0.883 ^§^
Body-mass index, median (IQR), kg/m^2^	24.1 (21.7–25.9)	24.3 (22.0–26.6)	0.235 *
Smoking, *n* (%)	2 (2)	6 (6)	0.279 ^‡^
Alcoholism, *n* (%)	6 (6)	12 (12)	0.138 ^†^
Constipation, *n* (%)	18 (18)	17 (17)	0.852 ^†^
Chronic laxative use, *n* (%)	5 (5)	4 (4)	>0.999 ^‡^
Previous abdominal/pelvic surgery, *n* (%)	44 (44)	39 (39)	0.473 ^†^
Indications for colonoscopy			
Screening	14 (14)	23 (23)	0.144 ^†^
Surveillance	86 (86)	77 (77)	0.144 ^†^

^†^ Chi-square test; ^‡^ Fisher’s exact test; * Student’s *t*-test; ^§^ Mann–Whitney U test. EGD, esophagogastroduodenoscopy; IQR, interquartile range.

**Table 2:** Outcomes of the bidirectional endoscopy.

Variable	EGD-First (*n* = 100)	Colonoscopy-First (*n* = 100)	*p* Value
Procedure time of bidirectional endoscopy, median (IQR), min	33.1 (27.9–36.7)	30.6 (27.2–33.5)	0.005 *
Outcomes of colonoscopy			
Cecal-intubation success, *n* (%)	100 (100)	100 (100)	>0.999 ^†^
Cecal-intubation time, median (IQR)	16.0 (12.1–19.1)	13.7 (11.3–16.2)	<0.001 *
Difficult colonoscopy, *n* (%)	30 (30)	10 (10)	0.001
Colonoscopy withdrawal time, median (IQR)	9.3 (7.7–11.4)	10.2 (8.5–11.7)	0.163 ^§^
Cleaning time, median (IQR), min	1.4 (1.0–1.9)	1.4 (0.9–1.8)	0.512 ^§^
Inspection time, median (IQR), min	6.7 (6.0–8.0)	7.5 (6.1–8.5)	0.060 ^§^
Polypectomy time, median (IQR), min	0.8 (0–1.7)	0.9 (0.3–1.9)	0.425 ^§^
Colonoscopy procedure time, median (IQR)	26.5 (20.4–27.0)	24.0 (20.4–27.1)	0.003 *
Volume of water infused during insertion (mL), median (IQR)	1040 (750–1272)	945 (750–1115)	0.038 ^§^
Volume of water aspirated during insertion (mL), median (IQR)	1100 (900–1500)	1000 (705–1300)	0.021 ^§^
Boston Bowel Preparation Scale, median (IQR)			
Right colon	3 (2–3)	3 (3–3)	0.105 ^§^
Transverse colon	3 (3–3)	3 (3–3)	0.684 ^§^
Left colon	3 (2–3)	3 (3–3)	0.013 ^§^
Total score	8.5 (8–9)	9 (8–9)	0.030 ^§^
Adenoma-detection rate, *n* (%)	45 (45)	49 (49)	0.571 ^†^
Polyp-detection rate, *n* (%)	71 (71)	77 (77)	0.333 ^†^
Number of cases requiring abdominal compression, *n* (%)	55 (55)	57 (57)	0.776 ^†^
Number of cases requiring change of position, *n* (%)	10 (10)	7 (7)	0.447 ^†^
Outcomes of EGD			
EGD procedure time, median (IQR)	3.2 (2.7–3.8)	3.1 (2.6–3.9)	0.360 ^§^
Patient tolerance during the EGD, median (IQR)			
Counts of gag	0 (0–0)	0 (0–0)	0.788 ^§^
Counts of cough	0 (0–0)	0 (0–0)	0.873 ^§^
EGD technical adequacy, median (IQR)	4 (4–4)	4 (4–4)	0.195 ^§^
EGD findings, *n* (%)			
Reflux esophagitis	23 (23)	29 (29)	0.333 ^†^
Gastritis	32 (32)	40 (40)	0.239 ^†^
Gastric ulcer	19 (19)	14 (14)	0.446 ^†^
Gastric polyp	26 (26)	27 (27)	0.873 ^†^
Gastric erosion	11 (11)	6 (6)	0.205 ^†^
Duodenal ulcer	6 (6)	4 (4)	0.516 ^†^

^†^ Chi-square test; * Student’s *t*-test; ^§^ Mann–Whitney U test. EGD, esophagogastroduodenoscopy; IQR, interquartile range.

**Table 3:** Comparison of propofol doses, recovery time, turnover time, sedation-related adverse events, and patients’ assessment of discomfort and satisfaction between the two study groups.

Variable	EGD-First (*n* = 100)	Colonoscopy-First (*n* = 100)	*p* Value
Propofol dose, median (IQR), mg			
Initial dose	100 (88–120)	100 (90–110)	0.176 ^§^
Dose used during EGD	0 (0–20)	0 (0–50)	0.049 ^§^
Loading dose before the second procedure	0 (0–20)	50 (0–77.8)	<0.001 ^§^
Dose used during colonoscopy	80 (50–110)	85 (50–120)	0.636 ^§^
Total dose	200 (180–250)	250 (200–300)	<0.001 ^§^
Recovery time, median (IQR), min	7.0 (4.0–10.7)	13.5 (10.0–17.0)	<0.001 ^§^
Turnover time of endoscopic room, median (IQR)	39.5 (34.7–45.5)	42.6 (38.3–49.3)	0.004
Sedation-related adverse events, *n* (%)			
Bradycardia	27 (27)	31 (31)	0.533 ^†^
Tachycardia	1 (1)	1 (1)	>0.999 ^†^
Oxygen desaturation	2 (2)	0 (0)	0.497 ^‡^
Hypertension	19 (19)	11 (11)	0.113 ^†^
Hypotension	6 (6)	2 (2)	0.279 ^‡^
Patients’ assessment of discomfort and satisfaction			
Pain ^^^, median (IQR)	0 (0–1)	0 (0–0)	0.004 ^§^
Bloating ^^^, median (IQR)	0 (0–2)	0 (0–0)	0.006 ^§^
Nausea ^^^, median (IQR)	0 (0–0)	0 (0–0)	0.951 ^§^
Dizziness ^^^, median (IQR)	0 (0–0)	0 (0–0)	0.830 ^§^
Sore throat ^^^, median (IQR)	0 (0–0)	0 (0–0)	0.325 ^§^
Patient satisfaction ^#^, median (IQR)	10 (10–10)	10 (9.7–10)	0.322 ^§^
Willingness to repeat, *n* (%)	95 (95)	89 (89)	0.248 ^‡^

^†^ Chi-square test; ^‡^ Fisher’s exact test; ^§^ Mann–Whitney U test; ^^^ Using a 10 cm visual analog scale (0: no discomfort, 10: most severe), ^#^ Scored 0 to 10 (0: not satisfied at all, 10: most satisfied); EGD, esophagogastroduodenoscopy; IQR, interquartile range.

## Data Availability

The data presented in this study are available on reasonable request.

## Supplementary Materials

The following supporting information can be downloaded at: https://www.mdpi.com/article/10.3390/jcm11051365/s1, Table S1: Studies evaluating discomforts post procedure or during the procedure with bidirectional endoscopy.

## Abbreviations

EGD: esophagogastroduodenoscopy; BDE, bidirectional endoscopy; ADR, adenoma-detection rate; BBPS, Boston Bowel Preparation Scale; IQR, interquartile range; WE, water exchange.

## References

[1] El-Serag H.B., Xu F., Biyani P., Cooper G.S. (2014). Bundling in medicare patients undergoing bidirectional endoscopy: How often does it happen?. Clin. Gastroenterol. Hepatol..

[2] Pongprasobchai S., Sriprayoon T., Manatsathit S. (2011). Prospective evaluation of gastrointestinal lesions by bidirectional endoscopy in patients with iron deficiency anemia. J. Med. Assoc. Thail..

[3] Zuckerman G., Benitez J. (1992). A prospective study of bidirectional endoscopy (colonoscopy and upper endoscopy) in the evaluation of patients with occult gastrointestinal bleeding. Am. J. Gastroenterol..

[4] Hsieh Y.H., Lin H.J., Tseng K.C. (2011). Which should go first during same-day bidirectional endosocopy with propofol sedation?. J. Gastroenterol. Hepatol..

[5] Cao Y., Yang J., Li J., Ao X., Zhang K.Y., Shen X.C., Chen D.F., Lan C.H. (2017). Comparison of procedural sequences in same-day painless bidirectional endoscopy: Single-center, prospective, randomized study. Dig. Endosc..

[6] Triadafilopoulos G., Aslan A. (1991). Same-day upper and lower inpatient endoscopy: A trend for the future. Am. J. Gastroenterol..

[7] Choi G.J., Oh H.C., Seong H.K., Kim J.W., Ko J.S., Kang H. (2020). Comparison of procedural sequence in same-day bidirectional endoscopy: A systematic review and meta-analysis. Korean J. Intern. Med..

[8] Chen S.W., Cheng C.L., Liu N.J., Tang J.H., Kuo Y.L., Lin C.H., Tsui Y.N., Lee B.P., Hung H.L. (2018). Which should go first during same-day upper and lower gastrointestinal endoscopydy. J. Gastroenterol. Hepatol..

[9] Tang J.H., Cheng C.L., Kuo Y.L., Tsui Y.N. (2016). Paired comparison of procedural sequence in same-day bidirectional endoscopy with moderate sedation and carbon dioxide insufflation: A prospective observational study. Saudi J. Gastroenterol..

[10] Carter D., Lahat A., Papageorgiou N.P., Goldstein S., Eliakim R., Bardan E. (2014). Comparison of procedural sequence in same-day consecutive bidirectional endoscopy using moderate sedation: A prospective randomized study. J. Clin. Gastroenterol..

[11] Choi J.S., Youn Y.H., Lee S.K., Choi J.Y., Kim H.M., Kim Y.J., Han K.J., Cho H.G., Song S.Y., Cho J.H. (2013). Which should go first during same-day upper and lower gastrointestinal endoscopy? A randomized prospective study focusing on colonoscopy performance. Surg. Endosc..

[12] Cho J.H., Kim J.H., Lee Y.C., Song S.Y., Lee S.K. (2010). Comparison of procedural sequences in same-day bidirectional endoscopy without benzodiazepine and propofol sedation: Starting at the bottom or the top. J. Gastroenterol. Hepatol..

[13] Laoveeravat P., Thavaraputta S., Suchartlikitwong S., Vutthikraivit W., Mingbunjerdsuk T., Motes A., Nugent K., Perisetti A., Tharian B., Islam S. (2020). Optimal sequences of same-visit bidirectional endoscopy: Systematic review and meta-analysis. Dig. Endosc..

[14] Hsieh Y.H., Tseng C.W., Hu C.T., Koo M., Leung F.W. (2017). Prospective multicenter randomized controlled trial comparing adenoma detection rate in colonoscopy using water exchange, water immersion, and air insufflation. Gastrointest. Endosc..

[15] Cadoni S., Sanna S., Gallittu P., Argiolas M., Fanari V., Porcedda M.L., Erriu M., Leung F.W. (2015). A randomized, controlled trial comparing real-time insertion pain during colonoscopy confirmed water exchange to be superior to water immersion in enhancing patient comfort. Gastrointest. Endosc..

[16] Hsieh Y.H., Koo M., Leung F.W. (2014). A patient-blinded randomized, controlled trial comparing air insufflation, water immersion, and water exchange during minimally sedated colonoscopy. Am. J. Gastroenterol..

[17] Cadoni S., Ishaq S., Hassan C., Falt P., Fuccio L., Siau K., Leung J.W., Anderson J., Binmoeller K.F., Radaelli F. (2021). Water-assisted colonoscopy: An international modified Delphi review on definitions and practice recommendations. Gastrointest. Endosc..

[18] Jia H., Koo M., Hsieh Y.H., Tseng C.W., Hu C.T., Zhang L., Dong T., Pan Y., Leung F.W. (2019). Factors associated with adenoma detection in propofol-sedated patients. J. Clin. Gastroenterol..

[19] Katzgraber F., Glenewinkel F., Fischler S., Rittner C. (1998). Mechanism of fatal air embolism after gastrointestinal endoscopy. Int. J. Leg. Med..

[20] Tseng C.W., Koo M., Hsieh Y.H. (2017). Cecal intubation time between cap-assisted water exchange and water exchange colonoscopy: A randomized-controlled trial. Eur. J. Gastroenterol. Hepatol..

[21] Tseng C.W., Koo M., Hsieh Y.H. (2020). Cecal intubation time between the use of one-channel and two-channel water exchange colonoscopy: A randomized controlled trial. J. Gastroenterol. Hepatol..

[22] Aldrete J.A. (1995). The post-anesthesia recovery score revisited. J. Clin. Anesth..

[23] Hassan C., Manning J., Gonzalez M.A.A., Sharma P., Epstein M., Bisschops R. (2020). Improved detection of colorectal adenomas by high-quality colon cleansing. Endosc. Int. Open.

[24] Jowhari F., Hookey L. (2020). Gastroscopy should come before colonoscopy using CO_2_ insufflation in same day bidirectional endoscopies: A randomized controlled trial. J. Can. Assoc. Gastroenterol..

[25] Barr J. (1995). Propofol: A new drug for sedation in the intensive care unit. Int. Anesthesiol. Clin..

[26] Chen S.C., Rex D.K. (2004). Review article: Registered nurse-administered propofol sedation for endoscopy. Aliment. Pharmacol. Ther..

[27] Baudet J.S., Diaz-Bethencourt D., Aviles J., Aguirre-Jaime A. (2009). Minor adverse events of colonoscopy on ambulatory patients: The impact of moderate sedation. Eur. J. Gastroenterol. Hepatol..

[28] Lee Y.C., Wang H.P., Chiu H.M., Lin C.P., Huang S.P., Lai Y.P., Wu M.S., Chen M.F., Lin J.T. (2006). Factors determining post-colonoscopy abdominal pain: Prospective study of screening colonoscopy in 1000 subjects. J. Gastroenterol. Hepatol..

[29] Sumanac K., Zealley I., Fox B.M., Rawlinson J., Salena B., Marshall J.K., Stevenson G.W., Hunt R.H. (2002). Minimizing postcolonoscopy abdominal pain by using CO_2_ insufflation: A prospective, randomized, double blind, controlled trial evaluating a new commercially available CO_2_ delivery system. Gastrointest. Endosc..

