# Supplementary material for: Comparison of Procedural Sequences in Sedated Same-Day Bidirectional Endoscopy with Water-Exchange Colonoscopy: A Randomized Controlled Trial
Source: J Clin Med. 2022 Mar 2;11(5):1365. doi: 10.3390/jcm11051365 (PMC8911281; doi:10.3390/jcm11051365)
Supplement: Supplementary file 1 [file jcm-11-01365-s001.zip › jcm-1580256-supplementary.pdf]

Supplementary Table S1. Studies evaluating discomforts post procedure or during the procedure with bidirectional endoscopy.

Study	Country	Patient number	Sedation regimen	Patient self-assessment discomfort (EGD-first vs. colonoscopy-first)
**Discomfort score post procedure**				
Current study	Taiwan	100/100	Propofol	Pain^: 0 (0–1) vs. 0 (0–0); *P* = 0.004<br>Bloating: 0 (0–2) vs. 0 (0–0); *P* = 0.006
Jowhari et al. (2020)[1]	Canada	48/47(air)<br>47/44(CO_2)	Midazolam/Fentanyl	Aggregate patient comfort score (0-46) in air group*:<br>12.8 ± 9.1 vs. 10.8 ± 5.4, *P* > 0.05
Carter et al. (2014)[2]	Israel	83/80	Midazolam/Meperidine	Pain*: 1.1 ± 2.3 vs. 1.5 ± 2.6; *P* = 0.261<br>Fullness*: 0.5 ± 1.5 vs. 0.4 ± 1.3; *P* = 0.648
Hsieh et al. (2011)[3]	Taiwan	89/87	Propofol/Meperidine	Pain$^{\#}$: 1.3 (0.0–5.5) vs. 0.8 (0.0–3.9); *P* = 0.104<br>Bloating$^{\#}$: 1.5 (0.0–5.8) vs. 1.2 (0.0–5.0); *P* = 0.283
Cao et al. (2017)[4]	China	106/103	Propofol/Remifentanil	Abdominal distension and cramps: 0.94% vs. 0%; *P* = 1.000
**Discomfort score during EGD and colonoscopy**				
Chen et al. (2018)[5]	Taiwan	60/60	Midazolam/Fentanyl	During EGD*: 0.6 ± 1.3 vs. 1.1 ±1.8; *P* = 0.07<br>During colonoscopy*: 0.8 ± 1.6 vs. 1.0 ± 1.8; *P* = 0.45
Choi et al. (2013)[6]	Korea	550/550	Midazolam + Propofol/Meperidine	During EGD*: 4.17 ± 2.44 vs. 4.75 ± 2.32; *P* = 0.005<br>During colonoscopy* : 4.45 ± 2.83 vs. 4.31 ± 2.58; *P* = 0.577.
Cho et al. (2010)[7]	Korea	40/40	Without sedation	During EGD*: 3.25 ± 2.15 vs. 5.23 ± 2.08; *P* < 0.001.<br>During colonoscopy* : 3.46 ± 2.25 vs. 3.73 ± 2.11; *P* = 0.593.
Tang et al. (2016)[8]	Taiwan	63/63(CO_2)	Midazolam/Fentanyl	During EGD*: 0.56 ± 0.96 vs. 1.16 ± 1.00; *P* < 0.001.<br>During colonoscopy* : 1.79 ± 2.31 vs. 1.81 ± 2.44; *P* = 0.382.
**Meta-analyses (Discomfort scores post procedure as well as during EGD and colonoscopy)**				

Laoveeravat et al. (2020)[9]		928/920	Include the studies of Hsieh et al, Chen et al, Choi et al., and Cho et al.	During EGD: SMD, –0.45; 95% CI, –0.80 to –0.09; *P* = 0.02; I2 = 73%.<br>During colonoscopy: SMD, –0.27; 95% CI, –0.58 to 0.03; P= 0.07; I2 = 62%.
Choi et al. (2020)[10]		928/930	Include the studies of Chen et al, Choi et al., and Cho et al.	Pain post procedure: SMD, 0.11; 95% CI, –0.45 to 0.68; *P* = 0.59; I^2 = 86%.<br>Fullness post procedure: SMD, 0.15; 95% CI, –0.07 to 0.36; *P* = 0.539; I^2 = 0%.<br>During EGD: MD: 0.64, 95% CI: 0.09–1.20, I^2 = 79.5%, P = 0.03.<br>During colonoscopy: MD: – 0.03, 95% CI: – 0.26 to 0.20, I2 =0%, P = 0.81.

^ median (IQR); *Mean ± SD; # Mean (95% credibility interval); SMD: standardized mean difference; CI: confidence interval; MD: mean difference

References:

1. Jowhari, F.; Hookey, L. Gastroscopy Should Come Before Colonoscopy Using CO2 Insufflation in Same Day Bidirectional Endoscopies: A Randomized Controlled Trial. J Can Assoc Gastroenterol 2020, 3, 120-126, doi:10.1093/jcag/gwy074.

2. Carter, D.; Lahat, A.; Papageorgiou, N.P.; Goldstein, S.; Eliakim, R.; Bardan, E. Comparison of procedural sequence in same-day consecutive bidirectional endoscopy using moderate sedation: a prospective randomized study. Journal of clinical gastroenterology 2014, 48, 236-240, doi:10.1097/MCG.0b013e3182a87e5f.

3. Hsieh, Y.H.; Lin, H.J.; Tseng, K.C. Which should go first during same-day bidirectional endoscopy with propofol sedation? J Gastroenterol Hepatol 2011, 26, 1559-1564, doi:10.1111/j.1440-1746.2011.06786.x.

4. Cao, Y.; Yang, J.; Li, J.; Ao, X.; Zhang, K.Y.; Shen, X.C.; Chen, D.F.; Lan, C.H. Comparison of procedural sequences in same-day painless bidirectional endoscopy: Single-center, prospective, randomized study. Digestive endoscopy: official journal of the Japan Gastroenterological Endoscopy Society 2017, 29, 330-337, doi:10.1111/den.12847.

5. Chen, S.W.; Cheng, C.L.; Liu, N.J.; Tang, J.H.; Kuo, Y.L.; Lin, C.H.; Tsui, Y.N.; Lee, B.P.; Hung, H.L. Optimal procedural sequence for same-day bidirectional endoscopy with moderate sedation: A prospective randomized study. J Gastroenterol Hepatol 2018, 33, 689-695, doi:10.1111/jgh.13971.

6. Choi, J.S.; Youn, Y.H.; Lee, S.K.; Choi, J.Y.; Kim, H.M.; Kim, Y.J.; Han, K.J.; Cho, H.G.; Song, S.Y.; Cho, J.H. Which should go first during same-day upper and lower gastrointestinal endoscopy? A randomized prospective study focusing on colonoscopy performance. Surgical endoscopy 2013, 27, 2209-2215, doi:10.1007/s00464-012-2741-2.

7. Cho, J.H.; Kim, J.H.; Lee, Y.C.; Song, S.Y.; Lee, S.K. Comparison of procedural sequences in same-day bidirectional endoscopy without benzodiazepine and propofol sedation: starting at the bottom or the top. J Gastroenterol Hepatol 2010, 25, 899-904, doi:10.1111/j.1440-1746.2009.06157.x.
8. Tang, J.H.; Cheng, C.L.; Kuo, Y.L.; Tsui, Y.N. Paired comparison of procedural sequence in same-day bidirectional endoscopy with moderate sedation and carbon dioxide insufflation: A prospective observational study. Saudi J Gastroenterol 2016, 22, 360-365, doi:10.4103/1319-3767.191140.
9. Laoveeravat, P.; Thavaraputta, S.; Suchartlikitwong, S.; Vutthikraivit, W.; Mingbunjerdsuk, T.; Motes, A.; Nugent, K.; Perisetti, A.; Tharian, B.; Islam, S.; et al. Optimal sequences of same-visit bidirectional endoscopy: Systematic review and meta-analysis. Digestive endoscopy : official journal of the Japan Gastroenterological Endoscopy Society 2020, 32, 706-714, doi:10.1111/den.13503.
10. Choi, G.J.; Oh, H.C.; Seong, H.K.; Kim, J.W.; Ko, J.S.; Kang, H. Comparison of procedural sequence in same-day bidirectional endoscopy: a systematic review and meta-analysis. Korean J Intern Med 2020, 35, 331-341, doi:10.3904/kjim.2019.319.
